# Prospects for Malaria Eradication in Sub-Saharan Africa

**DOI:** 10.1371/journal.pone.0001767

**Published:** 2008-03-12

**Authors:** Ricardo Águas, Lisa J. White, Robert W. Snow, M. Gabriela M. Gomes

**Affiliations:** 1 Instituto Gulbenkian de Ciência, Oeiras, Portugal; 2 Ecology and Epidemiology Group, Department of Biological Sciences, University of Warwick, Coventry, United Kingdom; 3 Malaria Public Health and Epidemiology Group, Centre for Geographic Medicine, Kenya Medical Research Institute, Nairobi, Kenya; 4 Centre for Tropical Medicine, University of Oxford, John Radcliffe Hospital, Headington, Oxford, United Kingdom; 5 Centro de Matemática e Aplicações Fundamentais, Universidade de Lisboa, Lisboa, Portugal; London School of Hygiene & Tropical Medicine, United Kingdom

## Abstract

**Background:**

A characteristic of *Plasmodium falciparum* infections is the gradual acquisition of clinical immunity resulting from repeated exposures to the parasite. While the molecular basis of protection against clinical malaria remains unresolved, its effects on epidemiological patterns are well recognized. Accumulating epidemiological data constitute a valuable resource that must be intensively explored and interpreted as to effectively inform control planning.

**Methodology/Principal Finding:**

Here we apply a mathematical model to clinical data from eight endemic regions in sub-Saharan Africa. The model provides a quantitative framework within which differences in age distribution of clinical disease are assessed in terms of the parameters underlying transmission. The shorter infectious periods estimated for clinical infections induce a regime of bistability of endemic and malaria-free states in regions of mesoendemic transmission. The two epidemiological states are separated by a threshold that provides a convenient measure for intervention design. Scenarios of eradication and resurgence are simulated.

**Conclusions/Significance:**

In regions that support mesoendemic transmission, intervention success depends critically on reducing prevalence below a threshold which separates endemic and malaria-free regimes.

## Introduction

Approximately 2.2 billion people are affected by *Plasmodium falciparum* in 86 endemic countries, resulting in about 515 million clinical cases worldwide and over 1 million fatalities in Africa every year [1], [2]. A characteristic of *P. falciparum* infections is the gradual acquisition of clinical immunity resulting from repeated exposures to the parasite [3]. However, any given infection produces a clinical outcome that depends on a combination of parasite, vector, host and environmental factors [4]. In highly endemic regions, both prevalence of infection and incidence of severe malaria are high in young children [5], [6] and pregnant women [7], whereas in older children and adults prevalence of infection is higher, while incidence of severe cases is lower. Thus, even after many exposures, humans are not resistant to infection, but develop clinical immunity that prevents symptomatic disease [5], [6], which is not solely dependent on host intrinsic age factors [8]. In low-endemic regions, however, malaria infection and morbidity show less age dependence, with infections being commonly symptomatic even in adults, due to less frequent immunologic stimulation [9].

Understanding the mechanism of acquisition of clinical immunity has become a classical problem in malaria research. Approaches to resolve this puzzle involve the development of comprehensive yet parsimonious mathematical models [10]–[15]. Here, we construct a model of malaria transmission incorporating immunological aspects into an epidemiological framework to infer how acquisition of immunity is translated into distinct age distributions of clinical malaria in different populations. We observe that acquisition of clinical immunity modulates the duration of infections, which in turn is responsible for the identification of transmission regimes where stable endemic equilibria coexist with stable disease-free solutions. This phenomenon, termed bistability, has important implication for public health which will be discussed.

The model and methodologies developed here are not bound to the data, in the type and form presented here, and can be used with other data sets if desired.

## Methods

### Transmission model

**Figure 1:**
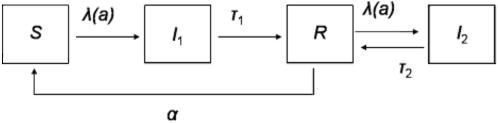
Model diagram representing the dynamics of malaria transmission. The compartments represent the following epidemiological classes: (S) completely susceptible individuals, either newborns, or those who have lost clinical immunity; (I1) clinical malaria cases resulting from infection of completely susceptible individuals; (R) individuals that have recovered from any infection and are clinically immune; (I2) asymptomatic malaria cases resulting from infection of clinically immune individuals.

The model is represented diagrammatically in Figure 1 and formalised by the system of differential equations:
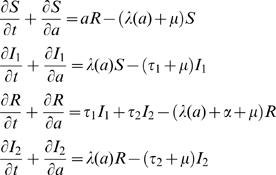
(1)


We consider that infections in immunologically naïve individuals (*S*) progress to a clinical form of malaria, requiring treatment, that we will henceforth call clinical malaria (*I*_1_). This group comprises both uncomplicated cases of clinical malaria which are treated without hospitalisation, and more severe cases requiring in-patient care. Based on the inference that immunity to severe malaria is acquired after one or two infections [13], we assume that recovery from infection eventually confers protection against clinical manifestations of disease, but not against infection *per se*. Hence, upon reinfection, a recovered individual (*R*) develops a non-clinical form of malaria, which we will refer to as asymptomatic infection (*I*_2_). While in the R compartment, individuals may lose their immunity, return to S and acquire another clinical infection (*I*_1_). Each variable represents a proportion of the total population. At the population level, patterns of occurrence of the two types of infection, *I*_1_ and *I*_2_, are intertwined and our aim is to identify mechanisms consistent with reported prevalence of hospitalised clinical malaria. We specify rates of recovery from infection as *τ*_1 _and *τ*_2_, respectively, and assume that protection wanes at a rate, *α*, unless boosted by repeated exposures. The force of infection, *λ*(*a*), that varies between populations according to the value of *λ*_0_, and includes age dependence in agreement with previous studies [9], is defined as:
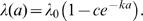
(2)


This function was derived from results of an entomological study, that correlates mosquito feeds with human body mass. The function is strictly increasing with age, with a minimum *λ*_0_(1−*c*) (at age zero) and upper limit *λ*_0_. Parameter *k* determines how steeply the force of infection increases with age, and *c* controls the amplitude of the increase. A summary measure of transmission is obtained by integrating the force of infection over age as
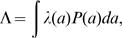
(3)where *P*(*a*) = *μ* e^−*μa*^, is the total population distributed over age and *μ* is the birth and death rate. Adopting standard assumptions, Λ is proportional to the frequency of infectious individuals, the proportionality constant being the transmission coefficient, *β* = Λ/(*I*_1_+*φI*_2_), where *φ* represents the relative infectivity of asymptomatic to clinical malaria infections.

The boundary conditions for system (1) at age *a* = 0 are *S*(*t*,0) = *μ* and *R*(*t*,0) = *I*_1_(*t*,0) = *I*_2_(*t*,0) = 0.

### The data

The datasets, from eight endemic sites, consist of retrospectively assembled data on paediatric admissions presenting from pre-defined catchment populations with clearly defined addresses to selected hospitals within 20km of their home, and where diagnosis was supported by detailed clinical examination and parasitology (see Appendix S1 for more details). Each dataset corresponds to a different region so that the ensemble represents a general perspective on the diversity of clinical malaria profiles, covering the spectrum of classical hypo to holoendemic transmission, typical of much of sub-Saharan Africa.

### Parameter estimation

Parameters are characterised into global (biological parameters intrinsic to the human host and, therefore, region-independent) and local (environmental parameters extrinsic to the human host and, therefore, region-specific). More specifically, we consider that rates of recovery and waning immunity are global and the force of infection is a local parameter [16]. As the full spectrum of clinical malaria infections is not captured solely by hospital admissions data, we introduce a rate of hospitalisation represented by an extra parameter, *η*, which is initially assumed equal to one and later on lowered for a sensitivity assessment. Model parameters are then estimated by fitting the prevalence of clinical malaria, *ηI*_1_, to age-stratified hospital admission rates, using a least squares minimisation method within Berkeley Madonna v8.3.6©. This is performed by running the age-structured model and fitting the output to all equally weighted datasets simultaneously (more details on the fitting method can be found in Appendix S1). As for the demographics, we assume a life expectancy of 50 years [17]. We have confirmed that increased mortality due to clinical malaria is negligible to our results and is, therefore, omitted.

### Equilibrium analysis

Equilibrium solutions are calculated by analytically solving the model without age structure using the global parameter values estimated previously. The transmission coefficients, *β*, are calculated by dividing the forces of infection integrated over age obtained from equation (3) by the sum, *I*_1_+*φI*_2_, in equilibrium conditions. We will represent the obtained equilibrium solutions as a function of transmission intensity, which is often represented in the form of the basic reproduction number.

### The basic reproduction number

The basic reproduction number, *R*_0_, is the average number of secondary infections generated by the introduction of a single infectious person into an otherwise naïve population. This theoretical number is calculated by multiplying the transmission coefficient, *β*, by the average duration of a primary infection, 1/(*τ*_1_+*μ*), to obtain *R*_0_ = *β*/(*τ*_1_+*μ*). We stress that although *R*_0_ can be indirectly calculated by manipulating parameters that can be estimated from an endemic equilibrium, this number is only meaningful when malaria is newly emerging in an immunologically naïve population. When transmission is endemic, a proportion *p* of infections occurs in clinically immune individuals. These infections are likely to show no clinical symptoms, remain untreated and transmit for a possibly different period, 1/(*τ*_2_+*μ*). In this case, the average number of infections generated by an infectious individual is *R*_0_ = *β*((1−*p*)/(*τ*_1_+*μ*)+*p*/(*τ*_2_+*μ*)).

## Results

Assuming, initially, the maximal hospitalisation rate, *η* = 1, the estimates obtained for the global parameters and the corresponding 95% confidence intervals are:
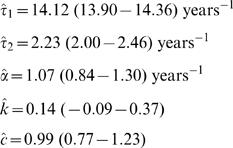
(4)


From (4) we can see that, on average, individuals with clinical malaria are infectious for a shorter time period (about 25 days) than those with an asymptomatic infection (circa 165 days). The duration of clinical immunity lasts for around 1 year and the age-dependent force of infection rises from 1% to 76% of its upper limit between ages 0 and 10 years. Under the initial assumption that clinical cases are as infectious as mild infections, *φ* = 1, we estimate region-specific forces of infection as listed in Table 1, alongside the corresponding values for the basic reproduction number.

**Table 1:** Estimated local parameters.

Region	*λ_0_* (95% c.i.)	Λ	*β*	R_0_
Bakau	0.14 (−0.09–0.37)	0.12	*NA*	*NA*
Foni Kansala	4.86 (4.63–5.09)	4.26	6.99	0.49
Sukuta	6.70 (6.47–6.93)	5.87	8.48	0.60
Mponda	14.96 (14.73–15.19)	13.10	15.52	1.10
Kilifi	19.87 (19.64–20.10)	17.40	19.77	1.40
Chonyi	47.21 (46.98–47.44)	41.35	43.66	3.08
Ifakara	50.16 (49.93–50.40)	43.94	46.25	3.27
Siaya	71.02 (70.79–71.25)	62.21	64.53	4.56

**Figure 2:**
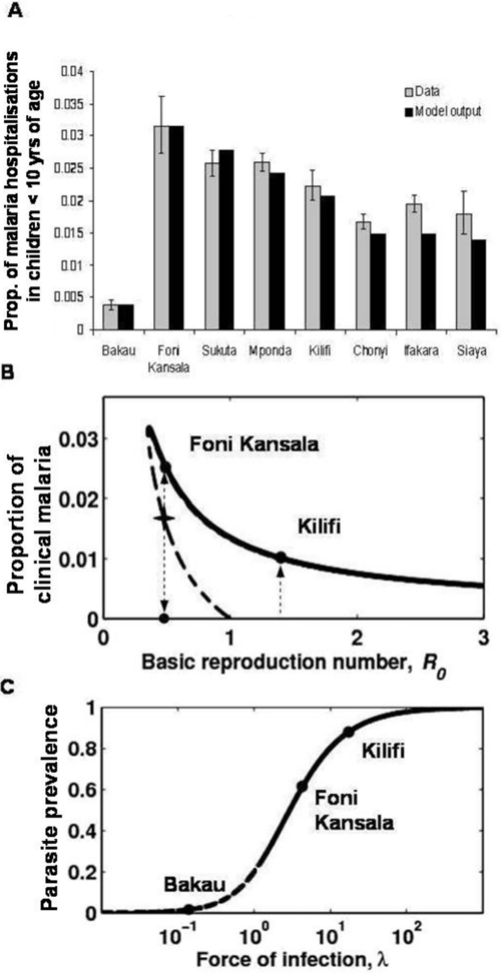
Endemic stability and bistability. (A) Prevalence of severe clinical malaria in children less than 10 years of age (grey) and model output (black). The error bars represent a 95% confidence interval. The eight regions are ranked by the estimated forces of infection. (B) Equilibrium proportion of clinical malaria over all ages in terms of R_0_. The full lines represent the stable solutions of the system of differential equations, whereas the dashed lines stand for unstable equilibria. Endemic equilibria are marked for two representative regions: Foni Kansala and Kilifi. Alternative disease-free equilibria for Foni Kansala is marked F. (C) Relationship between the force of infection (or entomological inoculation rate, EIR) and the parasite prevalence. The full line represents the stable solution of the system whereas the dashed line stands for an unstable equilibrium.

*NA*–The force of infection estimated for Bakau implies that this regions has unstable malaria transmission (as can be seen in Figure 2C), which renders inapplicable the method for calculation of *β* and consequently R_0_.

**Figure 3:**
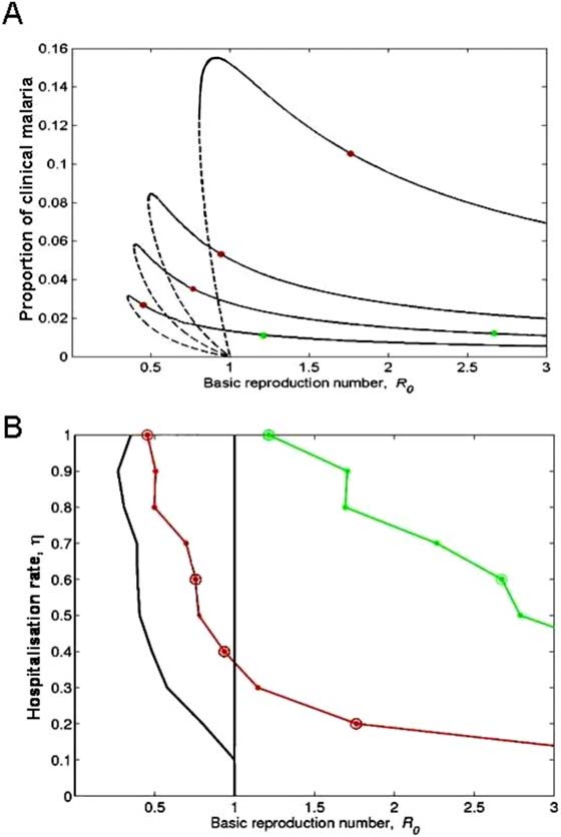
Sensitivity to hospitalisation rate, parameter η. (A) Equilibrium curves for different values of η (1, 0.6, 0.4, 0.2, from lower to higher peak). (B) Estimated basic reproduction numbers for different values of η. The black full line on the left shows the lower value of R_0_ for which an endemic equilibrium solution exists. The red and green dotted lines represent the values of R_0_, derived from the estimated forces of infection, for Foni Kansala and Kilifi, respectively. Circles highlight the values of ηcorresponding to the curves in (A).

**Figure 4:**
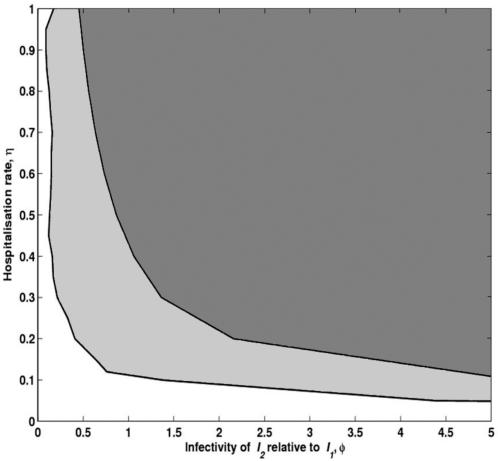
Parameter region for the existence of bistability. The grey area represents the region of parameters in which bistability is obtained. The darker grey area defines the conditions for bistability in Foni Kansala.

Figure 2 ranks the prevalence of clinical malaria and parasite prevalence by the associated transmissibility indices estimated under a baseline scenario (*η* = 1, *φ* = 1), whose robustness is challenged in Figures 3 and 4. Figure 2A represents the levels of malaria morbidity in children with less than 10 years of age, calculated as the prevalence of hospital admissions (from the data) and the value of *I*_1_ (from the model) ranked by the estimated forces of infection. We infer that prevalence of clinical malaria peaks at low to intermediate transmission to gradually decline as transmission increases. This phenomenon has been termed as endemic stability [18]. Figure 2B shows the equilibrium prevalence of clinical malaria in terms of the basic reproduction number. The peak of clinical malaria is again visible at low transmission, although this effect would be less pronounced for lower rates of waning immunity. More striking, however, is the result that the model supports two stable equilibria for a range of *R*_0_<1. Figure 2C represents the parasite prevalence in terms of the force of infection, confirming qualitative agreement with previous studies [14].

The existence of two stable equilibria for the same parameter values is named bistability. This is generated by a positive feedback mechanism that enhances transmission when endemicities are established [19], [20], which occurs here when recovery from a clinical infection is faster than from asymptomatic infections (*τ*_1_>*τ*_2_). This framework provides an identification of regimes where malaria control is sustainable. Endemic and disease-free states are separated by an unstable equilibrium that represents the critical prevalence below which the system converges to the disease-free state. The datasets considered in this study include regions that are representative of the various scenarios produced by the model. We consider three regions for illustration purposes. In hyperendemic regions (e.g Kilifi) the endemic equilibrium is the only stable solution, while in hypoendemic regions (e.g. Bakau), the system shows no stable endemic equilibrium consistently with a scenario of unstable transmission. In regions of mesoendemic transmission (e.g Foni Kansala), however, fixed parameter values can stably maintain a malaria-free state, or support transmission resulting in high levels of clinical disease. The outcome is determined by the initial conditions and can be manipulated by specially designed and carefully monitored interventions.

In Figure 3 we analyse the sensitivity of these findings to changes in the hospitalisation rate, *η*. Reducing the hospitalisation rate systematically contracts the bistability region, which persists until *η* reaches the proximity of 0.1. While lacking a rigorous estimate for this parameter, we cannot exclude the possibility that *η* is so low as to compromise the bistable behaviour. The robustness of the bistable regime is, nevertheless, reassured when we explore the relative infectivity of asymptomatic to clinical infection, *φ*. Figure 4 shows (in grey) the persistence of bistability throughout the (*φ*,*η*) parameter space. For concreteness, the parameter combinations where bistability is predicted for Foni Kansala are also marked (in dark grey). Recent empirical studies [21] suggest that asymptomatic infections are more infective than clinical infections, indicating that *φ*>1. Coincidentally, the analysis demonstrates that increasing *φ* enhances the bistability phenomenon favouring the realism of our findings.

## Discussion

Confronting a mathematical model with observed age profiles of clinical malaria, we obtain estimates for the parameters underlying the dynamics of transmission. In particular, rates of recovery from infection are obtained indicating that, on average, individuals with clinical malaria are infectious for a shorter time period than those with an asymptomatic infection. We attribute this mainly to behavioural factors. A clinical infection is likely to lead to administration of anti-malarials and faster recovery. This trend is consistent with estimates obtained from epidemiological observations of populations under treatment, where the average duration of infection was brought down from 270 to 14 days by administration of drugs [22].

Consistently with previous observations [6], [23] we describe a peak of prevalence of clinical malaria at low to intermediate transmission (Figure 2A). This is due to the phenomenon of endemic stability, which in our model is attributed to the dynamics of infection and immunity as follows. Under high force of infection, clinical immunity is maintained through repeated infections, and clinical cases become relatively rare. If the force of infection is reduced, clinical immunity wanes and the potential for occurrence of clinical cases, increases. Clinically immune individuals can either be reinfected and boost their immunity at rate Λ, or lose immunity at rate *α*. While Λ prevents the return to the susceptibility pool for clinical malaria, *α* has the opposite effect of generating more susceptibles. Since *α* is a global parameter, a peak in clinical disease is expected to occur as Λ varies across regions. This general model, applicable to malaria and other diseases (e.g. pertussis [24]), implies that interventions that reduce transmission should be combined with effective management of clinical cases to prevent any undesired increase in disease burden. These profound implications to control planning have motivated more detailed and highly controlled epidemiological studies [25]. Our results have no implications to this ongoing debate as the model used here does not take explicit account of specific syndromes of severe malaria nor mortality.

More relevant to malaria control, is the finding of bistable behaviour in regions of mesoendemic transmission, such as Foni Kansala and Sukuta. Bistability is generated by the establishment of longer infections in clinically immune individuals, in comparison to infections in those that are immunologically naïve. This asymmetry, which enhances transmission when endemicities are established, leads to the identification of conditions for sustainable malaria control. Interventions in the field have accumulated unsuccessful eradication attempts in regions of hyper to holoendemic transmission, such as the Garki district in Nigeria [26], in contrast with successful eradication initiatives taken place in areas of hypo or mesoendemic transmission, such as the highlands of Papua New Guinea (PNG), most of the South African territory, the midlevel area of Swaziland, and Sri Lanka [27]–[29]. In recent years, however, some of these areas (Sri Lanka and the highlands of PNG) have experienced malaria resurgence. This epidemiological change, which can be attributed to both the relaxation of control measures and population migration between these locations and areas where malaria is endemic [29], [30], is consistent with the regime *R*_0_<1 in Figure 2B, where either malaria free (horizontal branch) or endemic (higher branch) states are stable once established. Unstable malaria transmission is an epidemiological scenario that, while also falling into the *R*_0_<1 regime, represents regions (such as Bakau) where patterns of clinical malaria incidence are consistent with perturbations of the malaria-free state rather than a stable endemic state.

A practical tool for control planning comes from the observation that the two epidemiological states are separated by an unstable state (dashed branch) indicating a threshold for either eradication or resurgence. This threshold, expressed in terms of parasite prevalence, or other measures convenient to intervention design, is reduced if the typical duration of infection increases. Stability of the malaria-free state relies, therefore, on a system of early detection and response, whose purpose is to shorten the infectious period of parasite-positive individuals through antimalarial treatment. Shifts from disease-free to endemic states are usually triggered by a sufficiently intense migration from endemic regions, and can be prevented by early detection and treatment of malaria infections irrespective of symptoms [29]. Therefore, to achieve malaria eradication in areas of hypo and mesoendemic transmission, an integrated effort between those regions and their contiguous hyper and holoendemic regions is imperative. Conditions for eradication and resurgence are simulated in Appendix S1.

The model adopted here is consistent with previous models in malaria epidemiology [11], [12] and embodies plausible hypotheses about the natural history of malaria infection. Age dependence is especially elaborated so the model output can be adjusted to age-stratified datasets. As the model is intended to reproduce age profiles of annual hospital admissions, those transitions that do not directly affect the clinical malaria compartment are simplified. The most significant reductions refer to the explicit details of how immunity is maintained and the mosquito section of the transmission cycle. Although models often differ in the exact details of super- and re-infection, they are consensual in that immunity is boosted by repeated exposure. The originality of our procedure is the estimation of independent infection and recovery parameters for naïve and clinically immune individuals, indicating that the best fits to age profiles of clinical malaria are obtained when asymptomatic infections are greater contributors to transmission due to their larger infectious period. Other authors did not capture this behaviour because they imposed that asymptomatic infections were not infectious [11], [12]. Our model challenges this assumption, in agreement with the measured gametocyte densities [21] and the growing perception that mass administration of antimarial drugs should be an important component of malaria eradication strategies [31]–[33].

Mosquito population and infection dynamics is another classical topic in malaria research that has recently recovered attention from modellers [34]–[36]. Although we do not expect explicit details on this part of the cycle to impact qualitatively on the bistability mechanism, this should be carefully considered on any model extension intended to guide interventions in specific settings where malaria is seasonal.

Finally, the results presented here have important implications for the interpretation of measured indices of transmission. The basic reproduction number for malaria has recently been reformulated to consider heterogeneity in biting rates, resulting in an increase of *R*_0_ estimates in regions of holoendemic transmission by an order of magnitude [14]. Here we are concerned with the other end of the range and show the opposite effect: due to a backward bifurcation, the values of *R*_0_ estimated directly in mesoendemic scenarios may appear higher than the number of secondary infections that would be generated by the introduction of a single infectious person into an otherwise naïve population. For concreteness, we calculate, from the endemic equilibrium solutions, the proportion, *p*, of infections which occurs in clinically immune individuals, and obtain values between 0.95 and 0.99 depending on the region considered. As for the recovery rate, *τ*_2_, this has been estimated as approximately 6.3 times lower than *τ*_1_. Replacing these values in the formula above given in Methods, we would obtain an “apparent” *R*_0_≈6.3*β*/(*τ*_1_+*μ*), which is about 6 times higher than the “true” basic reproduction number. As a consequence, malaria eradication in those regions appears more viable than previously thought.

## Supporting Information

Appendix S1 (0.39 MB PDF)
