# Supplementary material for: Prospects for Malaria Eradication in Sub-Saharan Africa
Source: PLoS One. 2008 Mar 12;3(3):e1767. doi: 10.1371/journal.pone.0001767 (PMC2262141; doi:10.1371/journal.pone.0001767)
Supplement: Appendix S1 — (0.39 MB PDF) [file pone.0001767.s001.pdf]

Appendix S1

Prospects for sustainable malaria control in areas of mesoendemic transmission in sub-Saharan Africa

Ricardo Águas, Lisa J. White, Robert W. Snow, M. Gabriela M. Gomes*.

**To whom correspondence should be addressed. E-mail: ggomes@igc.gulbenkian.pt*

The data

Datasets from eight different endemic sites of sub-Saharan Africa were analysed. The clinical patterns and transmission intensity characteristics have been described previously for five sites [1]. Here we have included a further three sites where data were assembled using similar methodologies. The datasets consist of retrospectively assembled data on paediatric admissions presenting from pre-defined catchment populations with clearly defined addresses to selected hospitals within 20km of their home. Diagnosis was supported by detailed clinical examination and parasitology. Children were recorded as having severe malaria where the primary diagnosis for admission to hospital was recorded at discharge from the hospital or within death certificates if the child did not survive. For modelling purposes we defined all children hospitalized with malaria as a primary diagnosis as clinical malaria, although we accept that these might include some patients admitted merely for observation and would overlap with a group of ambulatory patients not admitted to hospital. Surveillance periods included complete years and ranged from one year in Tanzania to five years for Kilifi, Kenya. The information regarding hospital admissions was discriminated in months for the first year of life, and in years up to the 10th birthday. The purposive selection of catchment areas proximal to the hospital minimized as far as possible variations in the rates of malaria admission attributable to physical access and utilization between settings. Furthermore, the selection criteria based on residence enabled the attribution of temporally and spatially coincidental estimates of malaria endemicity, as defined by community-based childhood parasite prevalence rates covering the spectrum of classical hypoendemic to holoendemic transmission typical of much of sub-Saharan Africa.

Table S1. Characteristics of study data sources

	Bakau, The Gambia[1]	*Foni Kansala, The Gambia*[2]	*Sukuta, The Gambia*[1]	*Mponda, Malawi*[3]	*Kilifi, Kenya*[1]	*Chonyi, Kenya*[1]	*Siaya, Kenya*[1]	*Ifakara, Tanzania*[4]
Surveillance period	1.91-12.94 **(4 years)**	1.94-12.95 **(2 years)**	1.92-12.94 **(4 years)**	1.95-12.95 **(1 year)**	11.90-10.95 **(5 years)**	6.92-5.96 **(4 years)**	1.92-12.92 & 11.94-10.96 **(3 years)**	5.91-4.92 **(1 year)**
Total severe malaria admissions	**108**	**193**	**605**	**356**	**1358**	**766**	**719**	**144**
All cause malaria rate 0-9 years p.a.	**3.89** (108/27792)	**31.49** (193/6129)	**25.78** (605/23468)	**22.30** (356/15966)	**25.88** (1363/52675)	**16.66** (766/45967)	**17.95** (719/40064)	**19.51** (144/7380)
Median age months (IQR) of malaria admissions 0-9 years	**41.5** (23.5, 75.5)	**36** (24, 60)	**36** (22, 60)	**21** (11, 31)	**24** (12, 40)	**11** (6, 21)	**11** (5, 23)	**10** (6, 18)
Parasite ratio in childhood and approximate endemicity class	**2%** Hypoendemic[1]	**31%** Mesoendemic[5]	**37%** Mesoendemic[1]	**40%** Mesoendemic[6]	**49%** Meso-hyperendemic[1]	**74%** Hyper-holoendemic[1]	**83%** Holoendemic[1]	**90%** Holoendemic[7]

1. Data derived directly from [1].

2. Data collected in 1997 in collaboration with Dr Gisela Schneider from the WEC clinic at Sibanor, which had maintained a detailed clinical and laboratory surveillance of all pediatric admissions since 1990. Only patients presenting from Foni Kansala district were recorded in the retrospective review of admission data.

3. Data were re-assembled from a series reported in [2] and raw data were provided by Dr Terrie Taylor. Data were only included from a circumscribed address area representing the southern part of Mponda Terretorial Area of Mangochi District

4. Data collected during an earlier study [3] were re-analyzed to ensure that the clinical admission data and the parasitological data were spatially

coincidental (Idete and Lumeno wards)

5. Parasite prevalence data derived from [4] and (Umberto D'Alessandro, personal communication).

6. Parasite prevalence data derived from [5].

7. Parasite prevalence data obtained from [6] and (Tom Smith, personal communication).

Fitting method

The model was calibrated through the estimation of parameters both extrinsic and intrinsic to the human host. Forces of infection and rates of recovery and waning immunity were estimated by confronting the output prevalence of clinical malaria, I_1, with data from different countries in sub-Saharan Africa. As a first step to fit the model to the data we aggregated the data in such a way as to be comparable to the model output. As such, we calculated disease rates per age class by dividing the number of admissions by the number of individuals in the respective class. We then proceed to use a least squares minimisation method within the Berkeley Madonna v8.3.6© software to fit the model output of the I_1 variable to the data, affected by a hospitalisation rate, η. The method minimises the sums of the squares of residuals in I_1, which is the vertical difference between the model prediction and the data output at each age. We run the age-structured model and fit it to all equally weighted datasets simultaneously. The results of this process are displayed in Figure S1, while the fitted parameter values obtained for each region and for the global spectrum of endemicities are shown in the main text.

We used unweighted non-linear regression, which assumes a Gaussian distribution of the scatter and the same standard deviation for all points, although the datasets available are biased towards the points from one year of age onwards. The finer stratification of the first year of age results in smaller sample sizes, accounting for the larger error bars displayed in Figure S1, which suggests the use of a weighted non-linear regression [7].

We performed an alternative approach, deferring from using weighted least squares in favor of implementing a log likelihood maximization which assumes Poissonian regression. This method results in a better adjustment to the higher age classes where the

sample size is larger in detriment of the fit to the first year of age.

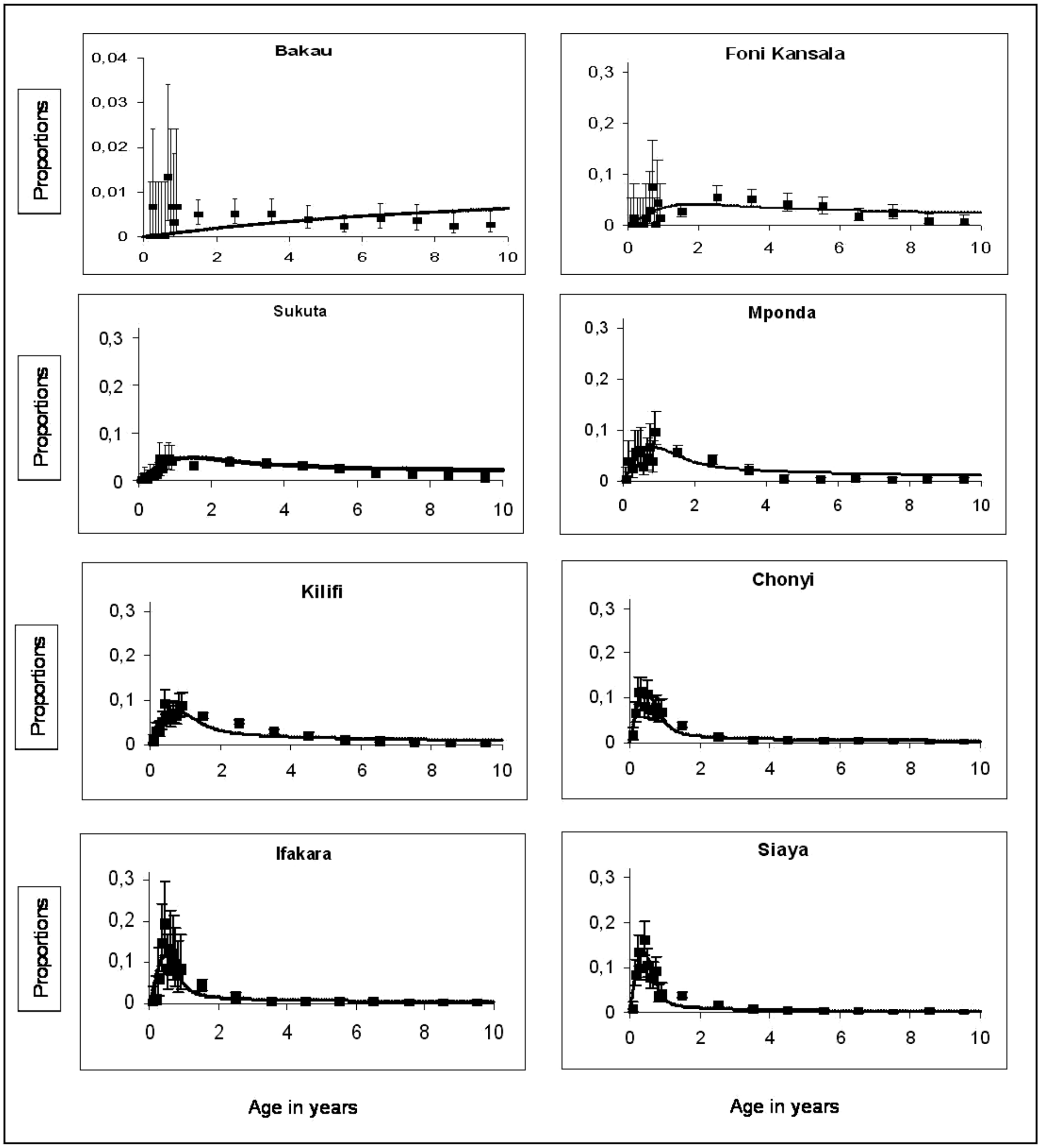

Figure S1. The age profiles of the proportion of hospital admissions at each age class for each region are represented by the squares, with the correspondent error bars depicted. The output of the model I_l variable that minimizes the root mean square of residuals, when the model is confronted with the data is shown by the curves.

Irrespective of the methods we obtain $\tau_1 > \tau_2$, which is the key to the results presented here. Indeed, under Poissonian regression the estimates are $\tau_1 = 5.24$ and $\tau_2 = 0.66$, translating into a ratio of 8, while with the Gaussian the estimates were are $\tau_1 = 14.12$ and $\tau_2 = 2.23$, corresponding to a ratio of 6.

The values for the initial guesses are chosen at random *a priori* from within the interval considered biologically plausible for each parameter. After a first fit, the resulting estimates are considered as an average of the first and second guesses. These values are then used as guesses for a new fit and are subsequently fine-tuned in an iterative manner. The ideal fit is the one that results in the computed least sum of square of residuals, and is a compromise between using a range of plausible parameter values and optimising the minimum least squares method result. The description of the age-dependent force of infection involves the three parameters, λ_0, k, and c, of which only the first is free to vary among locations. All the other parameters are constrained to have the same value for every location. We kept η equal to 1 in a first analysis. The value for this parameter is always fixed for a given fit, while all other parameters are being estimated.

We note that the fitting outcome is particularly sensitive to the value of τ_1, and less so to τ_2. More strict estimates of τ_2 would require the availability of data corresponding to the prevalence of mild infections (I_2) as well. By subsuming the vast range of clinical outcomes into two classes, the adopted model structure contributes to a high sensitivity of the results to the rate of waning immunity, α. Only the development of more constrained models, where a detailed system of acquired immunity is implemented, can limit these sensitivities. The trends presented here, however, can serve as a useful guide through the process of formulating and exploring tactic models and hypothesising mechanisms of

immunity in more detail.

Sensitivity analysis

To infer the importance of the non-estimated parameters over the model outcome, we performed multiple fits using different values for parameter η. Parameter η reflects the rate of under-reporting of clinical cases or percentage of clinical infections that account for hospitalisations. Tables S2 and S3 show the global and region-specific estimated parameters values, respectively, for a range of values of η.

Table S2. Estimated global parameters and 95% confidence intervals for different values of η.

η	0.9	0.8	0.7	0.6	0.5	0.4	0.3	0.2	0.1
τ_1	**14.72** (14.49-14.95)	**12.57** (12.34-12.79)	**10.69** (10.46-10.92)	**9.41** (9.18-9.64)	**8.28** (8.05-8.51)	**6.16** (5.93-6.39)	**4.22** (3.99-4.45)	**2.15** (1.92-2.38)	**0.52** (0.27-0.77)
τ_2	**1,05** (0.83-1.28)	**1.10** (0.88-1.33)	**1.37** (1.14-1.60)	**1.00** (0.78-1.23)	**0.70** (0.47-0.93)	**0.63** (0.40-0.85)	**0.48** (0.25-0.70)	**0.43** (0.20-0.65)	**0.29** (0.04-0.54)
α	**2.33** (2.11-2.56)	**2.07** (1.84-2.30)	**1.83** (1.60-2.06)	**2.30** (2.08-2.53)	**3.21** (2.98-3.44)	**2.98** (2.76-3.21)	**2.97** (2.75-3.20)	**2.06** (1.83-2.29)	**0.69** (0.44-0.93)
k	**0.09** (-0.14-0.32)	**0.11** (-0.12-0.34)	**0.09** (-0.14-0.32)	**0.09** (-0.14-0.32)	**0.11** (-0.12-0.33)	**0.11** (-0.12-0.34)	**0.13** (-0.10-0.36)	**0.16** (-0.07-0.39)	**0.63** (0.39-0.88)
c	**0.99** (0.77-1.22)	**1.00** (0.77-1.22)	**1.00** (0.77-1.22)	**1.00** (0.77-1.22)	**0.99** (0.77-1.22)	**0.99** (0.76-1.22)	**0.98** (0.75-1.21)	**0.97** (0.74-1.20)	**1.00** (0.75-1.25)

Table S3. Estimated region-specific parameters and 95% confidence intervals for different values of η.

	η = 0.9			η = 0.8			η =0.7		
	λ_0	β	R_0	λ_0	β	R_0	λ_0	β	R_0
Bakau	**0.18**<br>(-0.05-0.40)	9.20	0.62	**0.13**<br>(-0.10-0.36)	8.86	0.70	**0.17**<br>(-0.06-0.39)	7.84	0.73
Foni Kansala	**8.73**<br>(8.50-8.95)	7.45	0.50	**6.55**<br>(6.33-6.78)	6.26	0.50	**8.24**<br>(8.02-8.47)	7.45	0.70
Sukuta	**11.66**<br>(11.43-11.89)	9.36	0.64	**9.27**<br>(9.05-9.50)	8.08	0.64	**11.08**<br>(10.85-11.30)	9.31	0.87
Mponda	**26.64**<br>(26.42-26.87)	19.46	1.32	**20.71**<br>(20.49-20.94)	16.08	1.28	**25.43**<br>(25.21-25.66)	19.03	1.78
Kilifi	**34.99**<br>(34.76-35.22)	25.14	1.71	**28.06**<br>(27.84-28.29)	21.30	1.69	**33.08**<br>(32.85-33.30)	24.25	2.26
Chonyi	**89.19**<br>(88.96-89.42)	61.82	4.19	**71.00**<br>(70.77-71.23)	51.92	4.12	**86.06**<br>(85.83-86.29)	60.63	5.66
Ifakara	**94.72**<br>(94.49-94.95)	65.97	4.48	**71.89**<br>(71.66-72.12)	52.55	4.18	**85.13**<br>(84.90-85.35)	59.99	5.60
Siaya	**135.08**<br>(134.85-135.30)	93.59	6.35	**106.72**<br>(106.49-106.95)	77.42	6.15	**123.95**<br>(123.72-124.18)	86.68	8.09

	η = 0.6			η = 0.5			η =0.4		
	λ_0	β	R_0	λ_0	β	R_0	λ_0	β	R_0
Bakau	**0.15**<br>(-0.08-0.38)	7.11	0.75	**0.15**<br>(-0.08-0.38)	6.31	0.76	**0.13**<br>(-0.10-0.36)	5.03	0.81
Foni Kansala	**8.42**<br>(8.19-8.65)	7.15	0.76	**7.51**<br>(7.29-7.74)	6.44	0.78	**6.68**<br>(6.45-6.91)	5.78	0.94
Sukuta	**11.53**<br>(11.30-11.76)	9.18	0.97	**10.29**<br>(10.07-10.52)	8.31	1.00	**9.18**<br>(8.95-9.41)	7.48	1.21
Mponda	**26.46**<br>(26.23-26.68)	19.23	2.04	**23.41**<br>(23.18-23.64)	17.46	2.10	**21.16**<br>(20.94-21.39)	15.92	2.58
Kilifi	**35.24**<br>(35.02-35.47)	25.21	2.67	**31.48**<br>(31.25-31.71)	23.15	2.79	**28.57**<br>(28.34-28.80)	21.19	3.43
Chonyi	**94.23**<br>(94.00-94.45)	65.44	6.94	**87.89**<br>(87.66-88.12)	63.07	7.60	**81.01**<br>(80.78-81.23)	58.65	9.49
Ifakara	**93.48**<br>(93.25-93.71)	64.93	6.88	**85.32**<br>(85.09-85.55)	61.25	7.38	**77.68**<br>(77.46-77.91)	56.28	9.11
Siaya	**139.11**<br>(138.88-139.33)	96.09	10.19	**128.53**<br>(128.30-128.76)	91.87	11.07	**118.99**<br>(118.76-119.22)	85.82	13.89

	η = 0.3			η = 0.2			η =0.1		
	λ_0	β	R_0	λ_0	β	R_0	λ_0	β	R_0
Bakau	**0.12** (-0.10-0.36)	3.60	0.85	**0.06** (-0.17-0.28)	2.06	0.95	**0.03** (-0.22-0.27)	0.55	1.02
Foni Kansala	**5.53** (5.30-5.75)	4.86	1.15	**4.17** (3.94-4.40)	3.82	1.76	**2.03** (1.78-2.28)	2.06	3.79
Sukuta	**7.60** (7.37-7.83)	6.30	1.49	**5.85** (5.63-6.08)	5.04	2.33	**3.53** (3.28-3.78)	3.29	6.07
Mponda	**17.90** (17.67-18.13)	13.72	3.24	**14.55** (14.32-14.77)	11.53	5.32	**12.71** (12.46-12.95)	10.93	20.16
Kilifi	**24.35** (24.12-24.58)	18.42	4.34	**20.39** (20.17-20.62)	15.94	7.35	**25.40** (25.16-25.65)	21.52	39.69
Chonyi	**73.28** (73.05-73.50)	54.21	12.78	**73.80** (73.57-74.03)	56.33	25.98	**150.40** (150.16-150.65)	125.82	232.04
Ifakara	**67.97** (67.74-68.20)	50.33	11.87	**61.75** (61.52-61.97)	47.21	21.78	**69.00** (68.75-69.25)	57.89	106.77
Siaya	**107.22** (106.99-107.45)	79.06	18.65	**103.61** (103.39-103.84)	78.89	36.39	**195.74** (195.50-195.99)	163.65	301.82

The forces of infection estimated for each region, allowed us to test the effect of a parameter ϕ (relative infectivity of I_2 with respect to I_1) in equilibrium conditions. For this analysis we solved the model system of equations (described in the Methods of the main text) analytically taking into account ϕ embedded into the transmission coefficient: $\beta = \Lambda/(I_1 + \phi I_2)$. For η = 1 we obtain the equilibrium results depicted in Figure S2, given that $R_0 = \beta/(\tau_1 + \mu)$. It is evident from Figure S2, and as discussed in the main text, that I_2 type infections are extremely important for supporting high levels of prevalence of clinical malaria at very low levels of transmission. Decreasing the infectiousness of this type of infection below 1 (by decreasing ϕ) will reduce the potential endemic equilibrium, while increasing ϕ enhances the width of the bistable region.

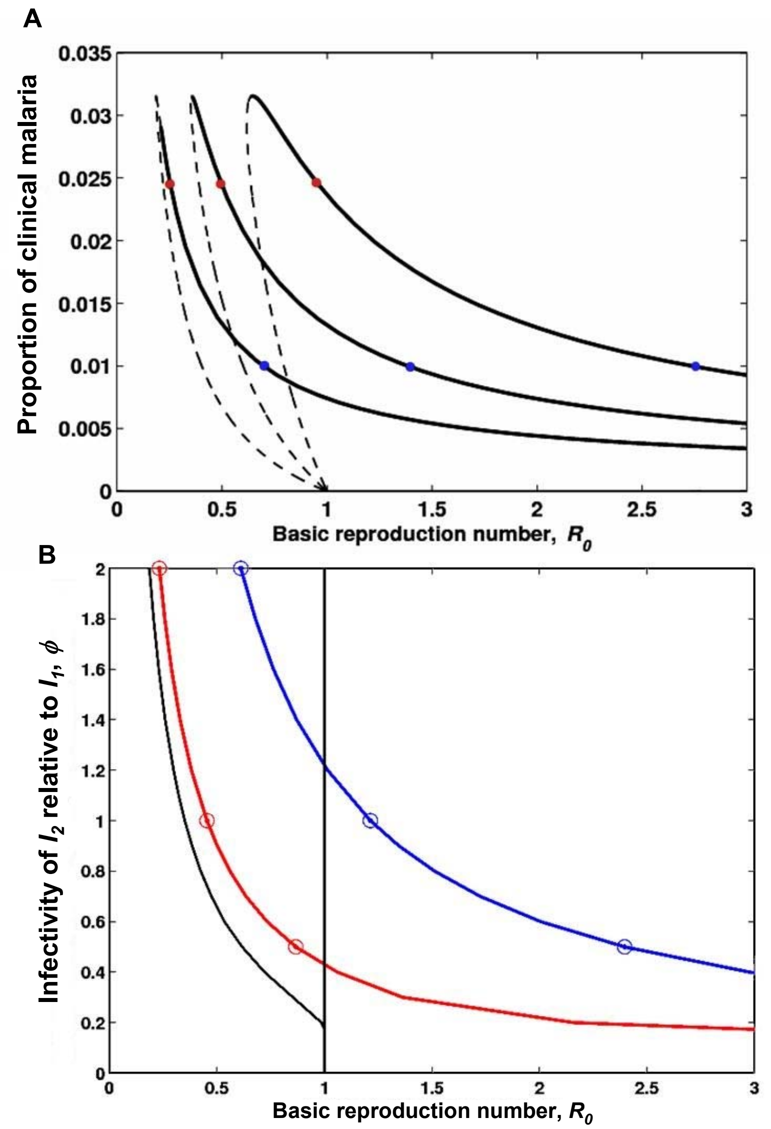

Figure S2. **(A)** Equilibrium curves for different values of ϕ (0.5, 1, and 2, from right to left). The full part of the curves represents the stable solutions at equilibrium, whereas the dashed part refers to unstable equilibria. **(B)** This diagram shows the values of ϕ for which bistability exists and how this parameter influences the value of R_0 for 2 regions. The black full line represents the minimum values of R_0 for which an endemic equilibrium solution exists, for each value of ϕ. The red and blue full lines refer to the values of R_0 estimated for Foni Kansala, and Kilifi, respectively, according to the value of ϕ. Circles highlight the values of ϕ corresponding to the curves in (A).

Simulating interventions

In a bistable system, convergence to either a malaria-free regime or sustained transmission resulting in high levels of clinical disease is determined by the initial conditions, and can be manipulated by specially designed interventions. We simulated several scenarios of practical interest to regions of mesoendemic transmission, such as Foni Kansala. For illustration purposes we show the results when η is equal to unity in Figure S3.

Figure S3(A,C) is concerned with intervention design showing that eradication is sustainable, but may be just missed, stressing the importance of tailoring the invested effort to the epidemiological context. We simulate antimalarial treatment of all infected individuals, regardless of severity of disease, by bringing the recovery rate, τ_2, up to the same value as τ_1. We chose to implement the extreme intervention of treating uncomplicated malaria as effectively as clinical malaria. Any therapeutic intervention will be less powerful than that simulated here but the qualitative behaviour will be maintained. Here, the intervention is implemented for a fixed time period and then halted. Maintaining the effort for 10 months and 10 days (dashed line) leads to sustained eradication, while intervening for 10 months and 9 days (full line) gives rise to resurgence and the original endemic equilibrium is re-established within a few years. Figure S3(B,D) deals with conditions for malaria to invade a malaria-free population. We simulate the introduction of individuals with uncomplicated malaria into a naive population. The grey area, with upper bound 0.017, represents the proportion of infections that can be introduced (maintaining the original rates τ_1 and τ_2) without establishing an endemic situation. An initial condition within this area proceeds to the

disease free equilibrium (dashed lines) while an initial prevalence above the grey area evolves to a endemic equilibrium sustaining mesoendemic transmission (full lines).

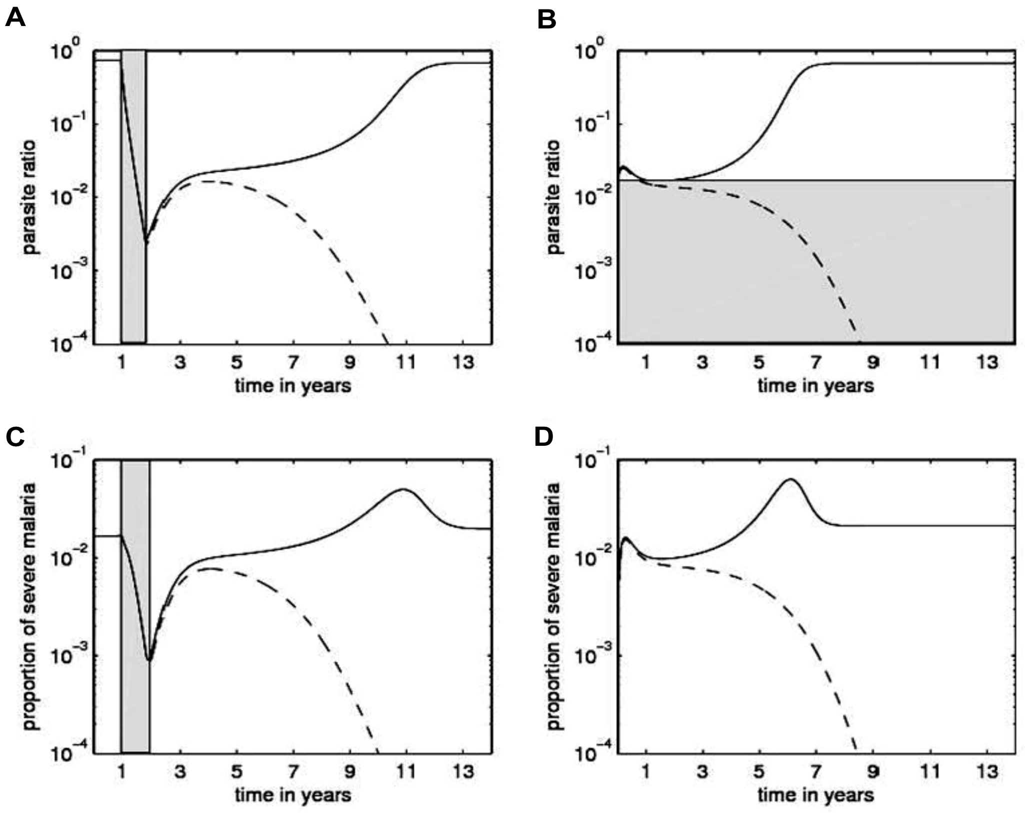

Figure S3. Threshold conditions for sustained control in areas of mesoendemic transmission. **(A,C)** simulation of an intervention that brings τ_2 up to the same value as τ_1. The intervention starts at the end of the first year and is maintained for 10 months and 9 days (full line) or 10 months and 10 days (dashed line). The grey area marks the time of intervention required for convergence to a disease free situation (sustained control). **(B,D)** simulation of the introduction of individuals with uncomplicated malaria into a completely susceptible population. The grey area, with upper bound 0.017, represents the proportion of infections that can be introduced (and cleared) without establishing sustained transmission (dashed lines). An initial prevalence above the grey area evolves to an endemic equilibrium (full lines). The simulations were performed with the parameters values estimated for Foni Kansala.

The duration of intervention necessary for eradication to be achieved as well as the proportion of new infections necessary for resurgence are greatly dependent on the level

of endemicity of the unstable solution for a given R_0 (depicted as a dashed line in Fig.2B of the main text). This endemicity level represents the threshold above which the system will converge to an endemic steady state, and below which the disease free situation is reached at equilibrium. The initial conditions are crucial in determining to which attractor the system will converge.

References

1. Snow RW. *et al*. (1997) Relation between severe malaria morbidity in children and level of Plasmodium falciparum transmission in Africa. Lancet. 349:1650-4.

2. Slutsker L, Taylor TE, Wirima J, Steketee RW (1994) In-hospital morbidity and mortality due to malaria associated severe anaemia in two areas of Malawi with different patterns of malaria infection. Trans R Soc Trop Med Hyg. 88:548-551.

3. Snow RW *et al*. (1994) Severe childhood malaria in two areas of markedly different falciparum malaria transmission in East Africa. Acta Tropica. 57:289-300.

4. Thomson MC *et al*. (1994) Malaria prevalence is inversely related to vector density in The Gambia, West Africa. Trans R Soc Trop Med Hyg. 88:638-43.

5. Yeudall F, Gibson RS, Kayira C, Umar E (2002) Efficacy of a multi-micronutrient dietary intervention based on hemoglobin, hair zinc concentrations, and selected functional outcomes in rural Malawian children. European Journal of Clinical Nutrition. 56:1176-85.

6. Smith T *et al*. (1993) Absence of seasonal variation in malaria parasitaemia in an area of intense seasonal transmission. Acta Tropica. 54:55-72.

7. Neter J, Kutner MH, Nachtsheim CJ, Wasserman, W (1996) Applied Linear

Statistical Models. Chicago: Irwin/McGraw-Hill. 1408p.
